# Prevalence of depression among Science students during the COVID-19 pandemic period in Riyadh, Saudi Arabia

**DOI:** 10.3389/fpsyt.2023.1286283

**Published:** 2023-11-09

**Authors:** Mahmoud Abdulrahman Mahmoud

**Affiliations:** Imam Muhammad ibn Saud Islamic University, Riyadh, Saudi Arabia

**Keywords:** depression, science students, Riyadh, Saudi Arabia, COVID-19

## Abstract

**Introduction:**

The present study aimed to examine the prevalence of depression among science students during the COVID-19 pandemic.

**Methods:**

A survey-based study was conducted on 521 science students at Al Imam Mohammad Ibn Saud Islamic University, Riyadh, Saudi Arabia, using Beck’s Depression Inventory (BDI), during the course of the semester.

**Results:**

Showed that the Most students were female (62.19%). Sadness symptoms were observed among 59.69% of the students. We found a 77.74% overall prevalence of depression among students. Most individuals reported moderate BDI (25.34%), but extremely severe BDI was also reported in 7.29% of individuals. The BDI scores were highly significantly correlated with gender, education, and field of specialty (< 0.001) based on different statistical tests. BDI scores were significantly associated with number of the demographic and academic variables (*p* < 0.05).

**Discussion:**

The study found significant symptoms of depression among students who displayed characteristics of depression during the COVID-19 pandemic. Therefore, students should undergo psychological counseling during difficult pandemic periods to prevent depression and mental stress.

## Introduction

One of the most prevalent and serious mental ailments affecting today’s youth is depression. It can last for at least two weeks and is characterized by a loss of interest and persistent sadness in things that people typically find enjoyable (1). Nearly 350 million individuals of all ages suffer from depression (2). The lifetime prevalence of anxiety and depression is currently estimated to be between 5 and 70% among adolescents and young adults worldwide (3). According to estimates, 18% of adolescents experience depressive symptoms, and both men and women are more likely to experience such symptoms as they age (4).

The most well-known disorders affect a variety of behaviors, such as concentration, motivation, self-worth assessment, anxiety, and depression (5). While training for their future jobs, university students make up a unique demographic group that must continually deal with and adapt to a variety of psychological problems stemming from both social and academic factors (6).

Numerous studies have suggested that students’ constant exposure to stressful situations, such as the burden of academic study in a broad field, parental pressure, fear of failure, difficulties in the job market, and susceptibility to various emotional outbursts, causes mental health problems (7, 8). Additionally, depression and anxiety have an impact on a student’s professional and personal attributes. Professional issues include lowered academic standards and integrity, a loss of empathy and morality, and an increase in medical blunders (8, 9).

This study was carried out during the time frame of the COVID-19 pandemic. To minimize the spread of COVID-19 in Saudi Arabia, the Ministry of Health (MOH) implemented a number of preventive strategies, including lockdowns. Ultimately, the majority of schools and universities were shut down, and online teaching techniques were adopted by the educational system. Numerous studies have examined the pandemic’s long- and short-term consequences on people’s psychological and social health (10). Some studies have shown that the COVID-19 pandemic seriously hampered people’s ability to behave normally and maintain their mental health (11, 12).

Therefore, this study explored the prevalence of depression based on Beck’s Depression Inventory (BDI) among science students studying at Imam Mohammad Ibn Saud Islamic University, Riyadh, Saudi Arabia, during the COVID-19 pandemic period. A study of a number from the associated factors with depression was carried out.

## Materials and methods

### Study setting and population

This cross-sectional study was conducted during the COVID-19 pandemic period in the years 2020–2021. The subjects were science students studying at the College of Science at Imam Muhammad Ibn Saud Islamic University, Riyadh, Kingdom of Saudi Arabia. Science students in this college are admitted directly after high school.

A cluster sampling method was used to select students. The questionnaires were sent online to the student leaders of each academic year, who disseminated the questionnaires to all students through different social media platforms. During this period, the students were given the opportunity to complete the questionnaire independently. A total of 521 students responded to the survey which represent more than 50% of the total number of College of Science students.

The ethical research committee of the Institutional Review Board (IRB) of Imam Mohammed Ibn Saud Islamic University (IMSIU), Riyadh, Saudi Arabia, approved this study. The students were informed of the purpose of the study, and their informed consent was later obtained.

### Questionnaire

For this study, a questionnaire corresponding to the BDI model (13) was adopted. The questionnaire consisted of 21 items, and responses were scored using four options (0, 1, 2, and 3). For every question, 0 represented the lowest level of depression, whereas 3 represented the maximum level of depression. At the end of the questionnaire, the students encouraged to seek help if they have any related symptoms or thoughts. Also, students’ orientation activity was done in collaboration with academic affairs of the faculty of Science with students participation as peer education.

The interpretation of the level of depression based on the BDI score is as follows:

**Table:** 

BDI score range	Level of depression
1–10	Normal
11–16	Mild mood disturbance
17–20	Borderline clinical depression
21–30	Moderate depression
31–40	Severe depression
Over 40	Extreme depression

### Data analysis

Data recorded in a predesigned form were entered into a Microsoft Excel spreadsheet. All entries were double-checked for possible typing errors. The demographic characteristics of the study sample were defined. Bivariate analysis was done to delineate factors associated with total and BDI scores using an independent samples *t*-test or analysis of variance (ANOVA), and multivariable liner regression as dependent variables. Multiple comparisons of BDI scores according to different academic years and specialties were conducted using Tukey’s *post hoc* test. Significance was set at *p* < 0.05. All calculations were done using IBM SPSS Statistics 25.

## Results

Table 1 shows the demographic and academic characteristics of the 521 science students from Riyadh, most of whom were Saudi nationals. Most students were female (62.19%). The mean age was 21.78 ± 2.61 years. Since most of the individuals were students below 23 years of age, most were unmarried (93.09%). The maximum number of students was in the third year (42.03%), and most students belonged to a biology specialty (47.60%).

**Table 1:** The demographic and academic characteristics of the students.

Variables	Group	Frequency (*N* = 521)	Percentage
Age (Mean ± SD)	-	21.78 ± 2.61	-
Gender	Male	197	37.81%
	Female	324	62.19%
Marital status	Single	485	93.09%
	Married	36	6.91%
Education	Prep	40	7.68%
	1st year	38	7.29%
	2nd year	112	21.50%
	3rd year	219	42.03%
	4th year	112	21.50%
Specialty	Prep year	39	7.49%
	Biology	248	47.60%
	Maths	84	16.12%
	Physics	84	16.12%
	Chemistry	66	12.67%

The shows the frequency distribution of BDI responses among the students. All BDI responses were gathered from the questionnaire used in this research. The main symptoms related to depression were: “I feel sad” (19.19%), “I am not particularly discouraged about the future” (52.98%), “I feel I have failed more than the average person” (64.88%), “I do not enjoy things the way I used to” (33.40%), “I feel guilty a good part of the time” (54.32%), “I feel I may be punished” (26.49%), “I am disappointed in myself” (25.72%), “I am critical of myself for my weaknesses or mistakes” (32.82%), “I have thoughts of killing myself, but I would not carry them out” (20.15%), “I cry more now than I used to” (16.51%), “I am slightly more irritated now than usual” (37.81%), “I am less interested in other people than I used to be” (33.59%), “I put off making decisions more than I used to” (31.29%), “I am worried that I am looking old or unattractive” (21.50%), “I have to push myself very hard to do anything” (33.01%), “I do not sleep as well as I used to” (48.56%), “I get tired more easily than I used to” (34.17%), “My appetite is not as good as it used to be” (27.83%), “I have lost more than ten pounds” (29.94%), “I am worried about physical problems like aches, pains, upset stomach, or constipation” (35.32%), and “I am less interested in sex than I used to be” (16.89%).

Table 2 shows the frequency distribution of the different categories of the BDI among the students. We found that most students reported moderate BDI (25.34%). Extremely severe BDI was also reported in 7.29% of the students.

**Table 2:** Frequency distribution of different categories of BDI among the students.

BDI categories	Frequency (*N* = 521)	Percentage
Normal BDI	116	22.26%
Mild BDI	109	20.92%
Moderate BDI	132	25.34%
Severe BDI	126	24.18%
Extremely severe BDI	38	7.29%

Table 3 shows a comparison of the mean BDI scores with different demographic and academic data. The BDI scores were highly significant relationship with gender, education, and specialty based on different statistical tests (< 0.001). The BDI scores of females were found to be 1.40-fold of the male score.

**Table 3:** Comparison of mean BDI scores with different demographic and academic data.

Variables	Group	BDI (Mean ± SD)	Statistical test	Value of *p*
Gender	Male	14.94 ± 10.23	*t*-test	<0.001
	Female	21.02 ± 11.24		
Marital status	Single	18.92 ± 11.40	*t*-test	0.075
	Married	16.11 ± 8.72		
Education	Prep	19.63 ± 11.37	ANOVA	<0.001
	1st year	22.11 ± 11.59		
	2nd year	22.33 ± 12.56		
	3rd year	17.88 ± 10.48		
	4th year	15.30 ± 9.96		
Specialty	Prep	19.97 ± 11.3	ANOVA	<0.001
	Biology	18.24 ± 10.54		
	Math	16.96 ± 9.58		
	Physics	24.7 ± 13.57		
	Chemistry	14.45 ± 9.66		

*The difference of mean is significant at the *p* < 0.05.

Table 4 shows the results of the multiple linear regression analysis for the comparison of the BDI scores as dependent variables, with demographic and academic data as the independent variables. We found that BDI scores based on backward elimination of the multivariable regression were significantly associated only with age and gender variables (*p* < 0.05).

**Table 4:** Multiple linear regression analysis for comparison of BDI scores with demographic and academic data.

Variables		Unstandardized coefficients		Standardized coefficients	*t*	Significance
		B	Std. error	Beta		
1	(Constant)	28.688	4.888		5.869	0.000
	Gender	5.042	1.009	0.217	4.997	0.000
	Age	−0.836	0.258	−0.194	−3.237	0.001
	Maritial	0.255	2.121	0.006	0.120	0.904
	Education	−0.425	0.535	−0.043	−0.794	0.428
	Speciality	0.511	0.417	0.054	1.225	0.221
2	(Constant)	28.635	4.864		5.888	0.000
	Gender	5.069	0.984	0.219	5.153	0.000
	Age	−0.822	0.231	−0.190	−3.565	0.000
	Education	−0.431	0.533	−0.043	−0.808	0.419
	Speciality	0.507	0.416	0.053	1.221	0.223
3	(Constant)	29.885	4.610		6.483	0.000
	Gender	5.096	0.983	0.220	5.186	0.000
	Age	−0.929	0.189	−0.215	−4.904	0.000
	Speciality	0.448	0.409	0.047	1.095	0.274
4	(Constant)	29.382	4.588		6.405	0.000
	Gender	5.166*	0.981	0.223	5.267	0.000
	Age	−0.874	0.183	−0.202	−4.784	0.000

^a^Dependent variable: BDI.

Table 5 shows the multiple comparisons of the BDI scores with different academic years assessed using Tukey’s *post hoc* test. The mean BDI score of the first-year students was significantly associated with the mean BDI score of the fourth-year students (*p* < 0.05), the mean BDI score of the second-year students was significantly associated with the mean BDI scores of the third- and fourth-year students (*p* < 0.05), the mean BDI score of the third-year students was significantly associated with the mean BDI score of the second-year students (*p* < 0.05), and the mean BDI score of the fourth-year students was significantly associated with the mean BDI scores of the first- and second-year students (*p* < 0.05).

**Table 5:** Multiple comparison of BDI scores with different academic year by Tukey *post hoc* test.

Academic year (I)	Comparative academic year (J)	Mean difference of BDI (I-J)	Std. error	Significance
Prep	1st year	−2.48026	2.49223	0.858
	2nd year	−2.70536	2.02650	0.669
	3rd year	1.74372	1.89174	0.888
	4th year	4.32143	2.02650	0.208
1st year	Prep	2.48026	2.49223	0.858
	2nd year	−0.22509	2.06542	1.000
	3rd year	4.22398	1.93337	0.187
	4th year	6.80169^*^	2.06542	0.009
2nd year	Prep	2.70536	2.02650	0.669
	1st year	0.22509	2.06542	1.000
	3rd year	4.44908^*^	1.27805	0.005
	4th year	7.02679^*^	1.47018	0.000
3rd year	Prep	−1.74372	1.89174	0.888
	1st year	−4.22398	1.93337	0.187
	2nd year	−4.44908^*^	1.27805	0.005
	4th year	2.57771	1.27805	0.259
4th year	Prep	−4.32143	2.02650	0.208
	1st year	−6.80169^*^	2.06542	0.009
	2nd year	−7.02679^*^	1.47018	0.000
	3rd year	−2.57771	1.27805	0.259

*The difference of mean is significant at the *p* < 0.05.

Table 6 shows multiple comparisons of the BDI scores with different specialties analyzed using Tukey’s *post hoc* test. The mean BDI score of students in a biology specialty was significantly associated with the mean BDI score of physics students (*p* < 0.05); the mean BDI score of students in a math specialty was significantly associated with the mean BDI score of physics students (*p* < 0.05); the mean BDI score of physics students was significantly associated with the mean BDI scores of biology, math, and chemistry students (*p* < 0.05); and the mean BDI score of chemistry students was significantly associated with the mean BDI score of physics students (*p* < 0.05).

**Table 6:** Multiple comparison of BDI score with different specialty by Tukey *post hoc* test.

Academic year (I)	Comparative academic year (J)	Mean difference of BDI (I-J)	Std. error	Significance
Prep	Biology	1.73646	1.87693	0.887
	Maths	3.01007	2.11129	0.611
	Physics	−4.72802	2.11129	0.167
	Chemistry	5.51981	2.20068	0.090
Biology	Prep	−1.73646	1.87693	0.887
	Maths	1.27362	1.37553	0.887
	Physics	−6.46448^*^	1.37553	0.000
	Chemistry	3.78336	1.50915	0.091
Maths	Prep	−3.01007	2.11129	0.611
	Biology	−1.27362	1.37553	0.887
	Physics	−7.73810^*^	1.68129	0.000
	Chemistry	2.50974	1.79226	0.628
Physics	Prep	4.72802	2.11129	0.167
	Biology	6.46448^*^	1.37553	0.000
	Maths	7.73810^*^	1.68129	0.000
	Chemistry	10.24784^*^	1.79226	0.000
Chemistry	Prep	−5.51981	2.20068	0.090
	Biology	−3.78336	1.50915	0.091
	Maths	−2.50974	1.79226	0.628
	Physics	−10.24784^*^	1.79226	0.000

*The difference of mean is significant at the *p* < 0.05.

**Figure 1:**
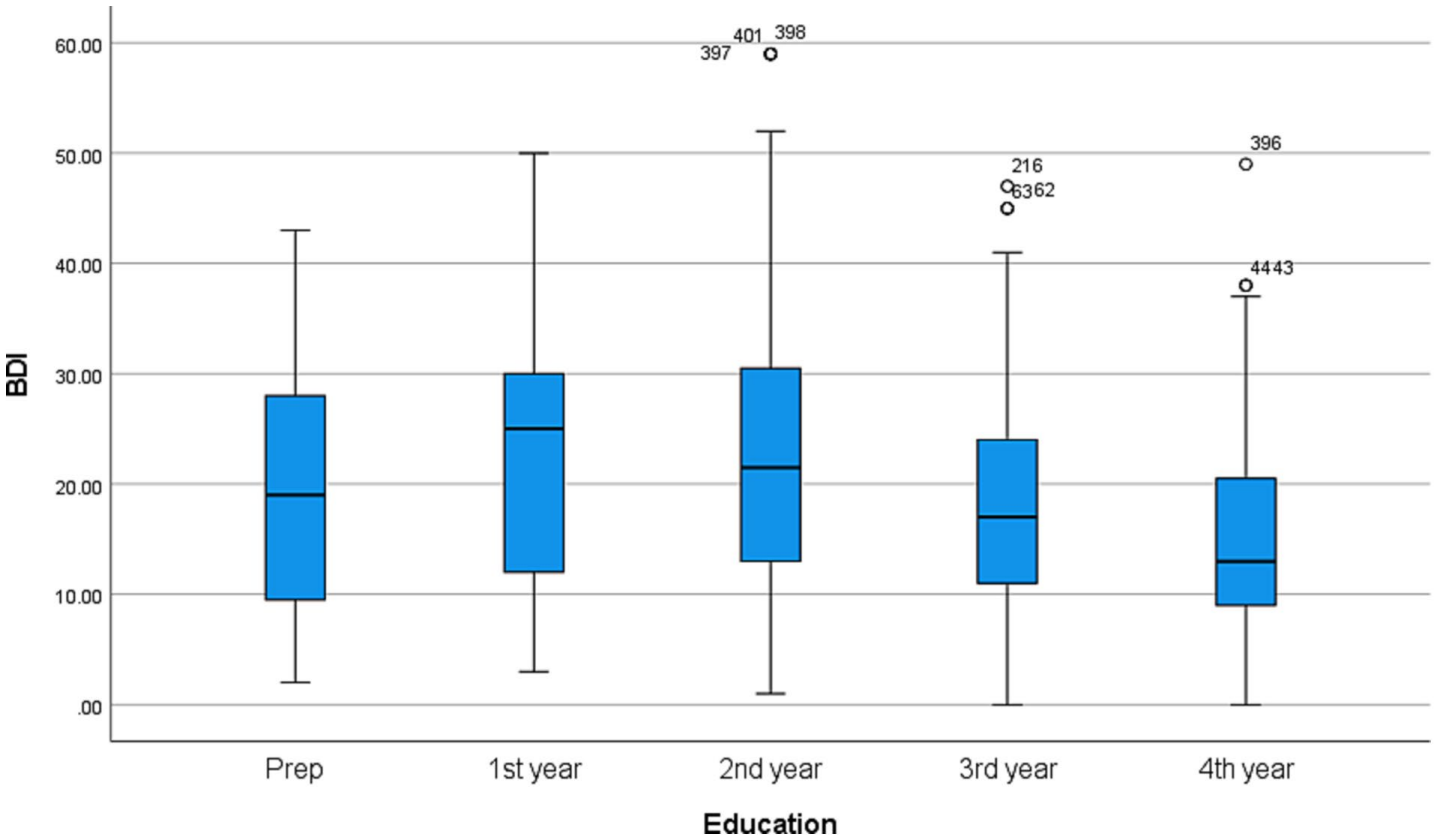
Box plot graph showing comparison of BDI scores with different academic year of the students.

**Figure 2:**
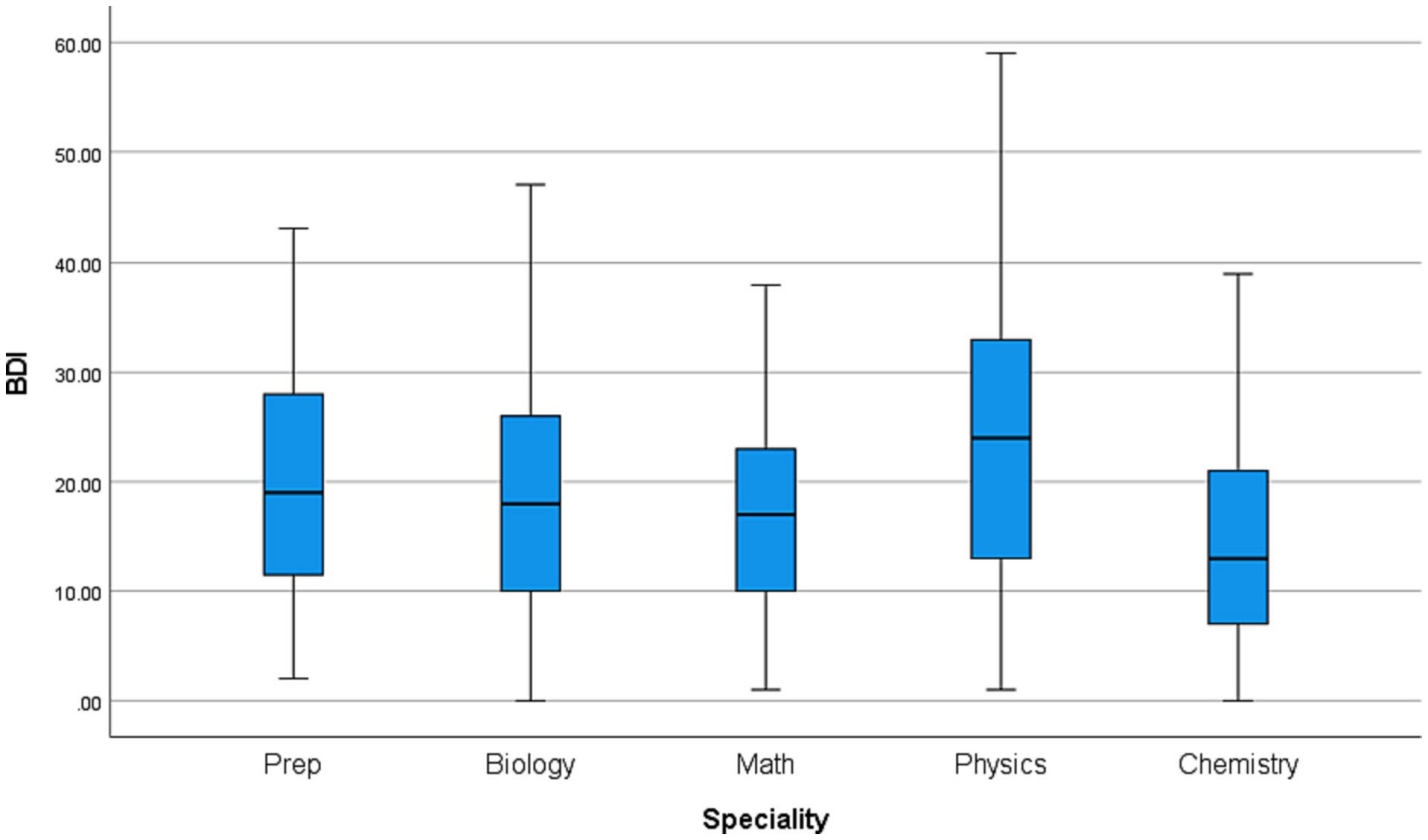
Box plot graph showing comparison of BDI scores with different specialty of the students.

Figure 1 shows a box plot graph comparing the BDI scores among students of different academic years. Figure 2 shows a box plot graph comparing the BDI scores of different students in different specialties.

## Discussion

The present study reports the prevalence of depression among science students during the COVID-19 pandemic. The findings revealed many symptoms pertaining to adverse mental health, which may eventually lead to depression. The reported depressive symptoms may be implicated in the mental stress associated with the burden of study and the psychological stress of the COVID-19 pandemic. We found a 77.74% overall prevalence of depression among students, in which extremely severe depression was found in 7.29% of the students. BDI scores were significantly associated with age and gender.

Suicidal conduct and thoughts are significantly predicted by depressive symptoms (14, 15). Depressive symptoms are associated with poorer academic performance, a higher risk of dropping out, and adverse health impacts (5, 16–18). We discovered that there was a high percentage of depression among university students, as we calculated various levels of depression in accordance with the BDI questionnaire. The majority fell into the mild to severe depression groups (20.92, 25.34, 24.18, and 7.29% with light, moderate, severe, and extremely severe depression, respectively).

In agreement with our findings, earlier research conducted abroad has shown that the majority of students suffer from mild to moderate depression (19, 20). Our findings, which are consistent with earlier studies on depression among young students, show that students under the age of 22 experience higher levels of depression (21–23).

The pandemic is an additional factor that may lead to depression, as many students experience increased stress levels, anxiety, and depressive symptoms as a result of altered university teaching methods, technological worries about online courses, and future employment. All these concerns have been observed in universities around the world (24).

Furthermore, numerous studies have examined how students were psychologically affected by the COVID-19 pandemic (25). A study from the USA (26) found that the COVID-19 outbreak increased stress and anxiety levels in students by 71%, which is consistent with some of the findings from our study.

Similar studies conducted in Vietnam and China also support our findings. Vietnamese female students were more likely to be depressed than male students (27), and Chinese females had more anxiety than males during the COVID-19 pandemic period. More female students in our study displayed depressive symptoms. Thus, the mental health of students must be closely monitored.

Earlier studies suggest that students are not always aware of accessible psychological services (7) and cannot recognize that they need treatment (28). Therefore, it is important to promote mental health awareness among students and to educate them about the forms of psychological support provided by the medical services of a university (29, 30).

## Conclusion

Significant symptoms of depression among science students during the COVID-19 pandemic were found in this study. The mental stress caused by the burden of study and the psychological stress resulting from the severe preventative measures imposed at the national level during the COVID-19 pandemic could have resulted in additional psychological stress and depressive symptoms. The implication of study findings is to review and promote the university students academic and psychological counseling with specific intervention plans during pandemics & disasters. The university needs to launch an awareness program to ensure that students are aware of the importance of mental health and to counter depression and mental stress among students, students should be encouraged to undergo psychological counseling during their study journey.

### Study limitations

as cross-sectional study, our result does not proving causation, our sample represents about one Faculty student from the university in an online scale method during the time of pandemic. We recommended further studies with inclusion of representative samples from all university students.

## Data availability statement

The raw data supporting the conclusions of this article will be made available by the authors, without undue reservation.

## Ethics statement

This study was approved by institutional ethical committee, IMSIU (98-2021) from Imam Muhammad Ibn Saud Islamic University. The studies were conducted in accordance with the local legislation and institutional requirements. The participants provided their written informed consent to participate in this study.

## Author contributions

MM: Writing – review & editing.

## References

[1] WHO Depression. (2017). World Health Organization. Available at: http://www.who.int/mental_health/management/depression/en/ (Accessed March 23, 2022)

[2] Pilania M Bairwa M Kumar N Khanna P Kurana H. Elderly depression in India: an emerging public health challenge. Australas Med J. (2013) 6:107–11. doi: 10.4066/AMJ.2013.1583, PMID: 23589734 PMC3626025

[3] Sahoo S Khess CR. Prevalence of depression, anxiety, and stress among young male adults in India: a dimensional and categorical diagnoses-based study. J Nerv Ment Dis. (2010) 198:901–4. doi: 10.1097/NMD.0b013e3181fe75dc 21135643

[4] Saluja G Iachan R Scheidt PC Overpeck MD Sun W Giedd JN. Prevalence of and risk factors for depressive symptoms among young adolescents. Arch Pediatr Adolesc Med. (2004) 158:760–5. doi: 10.1001/archpedi.158.8.760, PMID: 15289248

[5] Andrews B Wilding JM. The relation of depression and anxiety to life-stress and achievement in students. Br J Psychol. (2004) 95:509–21. doi: 10.1348/0007126042369802, PMID: 15527535

[6] Uehara T Takeuchi K Kubota F Oshima K Ishikawa O. Annual transition of major depressive episode in university students using a structured self-rating questionnaire. Asia Pac Psychiatry. (2010) 2:99–104. doi: 10.1111/j.1758-5872.2010.00063.x

[7] Eisenberg D Gollust SE Golberstein E Hefner JL. Prevalence and correlates of depression, anxiety, and suicidality among university students. Am J Orthopsychiatry. (2007) 77:534–42. doi: 10.1037/0002-9432.77.4.534 18194033

[8] Tabalipa FDO De Souza MF Pfützenreuter G Lima VC Traebert E Traebert J. Prevalence of anxiety and depression among medical students. Revista Brasileira De Educação Médica. (2015) 39:388–94. doi: 10.1590/1981-52712015v39n3e02662014

[9] Dyrbye LN Thomas MR Shanafelt TD. Medical student distress: Causes, consequences, and proposed solutions. Mayo Clin Proc. (2005) 80:1613–22. doi: 10.4065/80.12.1613 16342655

[10] Machado DB FJO A CSS T Rocha AS Castro-de-Araujo LF Singh A. Effects of COVID-19 on anxiety, depression and other mental health issues: a worldwide scope review. Res. Square. (2020). doi: 10.21203/rs.3.rs-58186/v1

[11] Loades ME Chatburn E Higson-Sweeney N Reynolds S Shafran R Brigden A. Rapid systematic review: the impact of social isolation and loneliness on the mental health of children and adolescents in the context of COVID-19. J Am Acad Child Adolesc Psychiatry. (2020) 59:1218–39.e3. doi: 10.1016/j.jaac.2020.05.009, PMID: 32504808 PMC7267797

[12] Gijzen M Shields-Zeeman L Kleinjan M Kroon H van der Roest H Bolier L. The bittersweet effects of COVID-19 on mental health: results of an online survey among a sample of the Dutch population five weeks after relaxation of lockdown restrictions. Int J Environ Res Public Health. (2020) 17:9073. doi: 10.3390/ijerph17239073, PMID: 33291765 PMC7730169

[13] Beck AT Steer RA Garbin MG. Psychometric properties of the beck depression inventory: twenty-five years of evaluation. Clin Psychol Rev. (1988) 8:77–100. doi: 10.1016/0272-7358(88)90050-5

[14] Cukrowicz KC Schlegel EF Smith PN Jacobs MP Van Orden KA Paukert AL. Suicide ideation among college students evidencing subclinical depression. J Am Coll Heal. (2011) 59:575–81. doi: 10.1080/07448481.2010.483710, PMID: 21823951 PMC5022368

[15] Nam B Hilimire MR Jahn D Lehmann M DeVylder JE. Predictors of suicidal ideation among college students: a prospective cohort study. Soc Work Ment Health. (2018) 16:223–37. doi: 10.1080/15332985.2017.1380742

[16] Hysenbegasi A Hass SL Rowland CR. The impact of depression on the academic productivity of university students. J Ment Health Policy Econ. (2005) 8:145–51. PMID: 16278502

[17] Stallman HM. Psychological distress in university students: a comparison with general population data. Aust Psychol. (2010) 45:249–57. doi: 10.1080/00050067.2010.482109

[18] Dendle C Baulch J Pellicano R Hay M Lichtwark I Ayoub S. Medical student psychological distress and academic performance. Med Teach. (2018) 40:1257–63. doi: 10.1080/0142159X.2018.1427222 29355074

[19] Waqas A Rehman A Malik A Muhammad U Khan S Mahmood N. Association of ego defense mechanisms with academic performance, anxiety and depression in medical students: a mixed methods study. Cureus. (2015) 7:1–7. doi: 10.7759/cureus.337 PMC4627837 26543695

[20] Singh MM Gupta M Grover S. Prevalence & factors associated with depression among school going adolescents in Chandigarh, North India. Indian J Med Res. (2017) 146:205–15. doi: 10.4103/ijmr.IJMR_1339_15, PMID: 29265021 PMC5761030

[21] Piumatti G. Motivation, health-related lifestyles and depression among university students: a longitudinal analysis. Psychiatry Res. (2017) 260:412–7. 29253806 10.1016/j.psychres.2017.12.009

[22] Burdzovic AJ Brunborg GS. Depressive symptomatology among Norwegian adolescent boys and girls: the patient health Questionnaire-9 (PHQ-9) psychometric properties and correlates. Front Psychol. (2017) 8:887. doi: 10.3389/fpsyg.2017.00887, PMID: 28642720 PMC5462997

[23] Ngasa SN Sama CB Dzekem BS Nforchu KN Tindong M Aroke D. Prevalence and factors associated with depression among medical students in Cameroon: a cross-sectional study. BMC Psychiatr. (2017) 17:216. doi: 10.1186/s12888-017-1382-3 PMC5466797 28599624

[24] Aristovnik A Ker Žiˇc D Ravšelj D Toma Ževiˇc N Umek L. Impacts of the COVID-19 pandemic on life of higher education students: a global perspective. Sustainability. (2020) 12:8438. doi: 10.3390/su12208438

[25] Cao W Fang Z Hou G Han M Xu X Dong J. The psychological impact of the COVID-19 epidemic on college students in China. Psychiatry Res. (2020) 287:112934. doi: 10.1016/j.psychres.2020.112934, PMID: 32229390 PMC7102633

[26] Son C Hegde S Smith A Wang X Sasangohar F. Effects of COVID-19 on college students’ mental health in the United States: interview survey study. J Med Internet Res. (2020) 22:e21279. doi: 10.2196/21279, PMID: 32805704 PMC7473764

[27] Nguyen DT Wright EP Dedding C Pham TT Bunders J. Low self-esteem and its association with anxiety, depression, and suicidal ideation in Vietnamese secondary school students: a cross-sectional study. Front. Psychiatry. (2019) 10:698. doi: 10.3389/fpsyt.2019.00698 PMC6777005 31611825

[28] Thomas SJ Caputi P Wilson CJ. Specific attitudes which predict psychology students’ intentions to seek help for psychological distress. J Clin Psychol. (2013) 70:273–82. doi: 10.1002/jclp.22022 23818259

[29] Henning K Ey S Shaw D. Perfectionism, the impostor phenomenon and psychological adjustment in medical, dental, nursing and pharmacy students. Med Educ. (1998) 32:456–64. doi: 10.1046/j.1365-2923.1998.00234.x, PMID: 10211285

[30] Huang L Lei W Xu F Liu H Yu L. Emotional responses and coping strategies in nurses and nursing students during Covid-19 outbreak: a comparative study. PLoS One. (2020) 15:e0237303. doi: 10.1371/journal.pone.0237303 32764825 PMC7413410

